# Nup153 and TPR/Megator Interact with TREX-2 Subunits and Are Essential for TREX-2-Dependent Nuclear Export of *hsp70* mRNA in *Drosophila*

**DOI:** 10.3390/ijms26178595

**Published:** 2025-09-04

**Authors:** Yulia Vdovina, Julia Nikolenko, Anastasia Orlova, Anna Glukhova, Maria Kurshakova, Savva Fet, Anna Tvorogova, Pyotr Tyurin-Kuzmin, Anton Golovnin, Sofia Georgieva, Daria Kopytova

**Affiliations:** 1 Engelhardt Institute of Molecular Biology, Russian Academy of Sciences, 119991 Moscow, Russia; yuvdov2020@gmail.com (Y.V.); julia.v.nikolenko@gmail.com (J.N.); chipushka@gmail.com (A.O.); anyapochta6@gmail.com (A.G.); kursha@mail.ru (M.K.); fetsavva@gmail.com (S.F.); sofia.georgieva2021@gmail.com (S.G.); 2 Center of Genomic Sciences, Institute of Gene Biology, Russian Academy of Science, 119334 Moscow, Russia; annatvor@mail.ru; 3 Faculty of Medicine, Medical Research and Educational Institute, Lomonosov Moscow State University, 119991 Moscow, Russia; tyurinkuzmin.p@gmail.com; 4 Institute of Gene Biology, Russian Academy of Science, 119334 Moscow, Russia; agolovnin@mail.ru

**Keywords:** mRNA export, TREX-2, TPR, Nup153

## Abstract

The TREX-2 complex is conserved from yeast to humans and is responsible for mRNA export from the nucleus to the cytoplasm. In yeast and humans, the TPR and Nup153 nucleoporins of the nuclear pore complex are involved in TREX-2-dependent mRNA export, but data on their involvement in this process is rather controversial. In the present work, we have studied the role of TPR and Nup153 in the TREX-2-dependent export of *hsp70* mRNA in *Drosophila*. We have shown that Nup153 and TPR are required for the TREX-2-dependent export of *hsp70* mRNA, and their knockdown leads to mRNA accumulation in the cell nucleus. We have also demonstrated that Nup153 knockdown leads to TPR relocation to the nucleoplasm. Both nucleoporins are required for TREX-2 subunits’ association with the nuclear pore. Nup153 depletion leads to the TREX-2 subunits’ relocation from the nuclear pore to the nucleoplasm. The depletion of TPR leads to PCID2 relocation to the nucleoplasm and Xmas-2 retention at the nuclear pore and does not affect ENY2 redistribution. The TREX-2 subunits form several contacts with Nup153 and TPR. Hence, both nucleoporins are involved in the interaction with TREX-2 and TREX-2-dependent export in *Drosophila*.

## 1. Introduction

In order to reach the protein synthesis sites in the cytoplasm, newly synthesized mRNA should be exported from the nucleus through the nuclear pore. The evolutionary conserved TREX-2 complex is one of the key players in this process [1,2,3,4,5,6,7]. It was previously shown that the complex is attached to the nuclear pore and participates in “gene gating”, where it is required for the docking of actively transcribed transcription loci near the nuclear pore [1,3,6,8,9]. In *Drosophila*, TREX-2 consists of the Xmas-2 (hGANP/ySac3), PCID2 (hPCID2/yThp1), ENY2 (hENY2/ySus1) and Sem1p (hDSS1/ySem1p) proteins [10]. Xmas-2 is the main scaffold subunit of the complex, with its N-terminus interacting with PCID2 and the C-terminus interacting with the two ENY2 molecules. Sem1p interacts with PCID2 and is believed to stabilize its structure [2,11].

Depletion of the subunits of this complex leads to bulk mRNA retention in the nucleus. It has been demonstrated that TREX-2 is responsible for the export of the major part of mRNA in yeast [1,12], while in humans, TREX-2 is involved in the export of mRNAs encoding specific subsets of mRNAs linked to transcriptionally active genes [13,14,15]. In *Drosophila*, the knockdown of TREX-2 subunits leads to the retention of bulk mRNA in the nucleus [3,10]. It has also been shown that TREX-2 is responsible for the export of the *hsp70* and *ras2* mRNAs in *Drosophila* [3,10]. Proper *hsp70* mRNA export into the cytoplasm is necessary for the correct expression of the *hsp70* gene and, therefore, is essential for cell survival under stress conditions.

TREX-2 complex subunits are located in the nucleus and are stably associated with the nuclear pores at the nuclear periphery [3,16]. In *Drosophila*, the PCID2 subunit of the TREX-2 complex is also localized in the cytoplasm of the cell, where it is associated with polysomes [17,18]. In the cytoplasm, PCID2 exists independently of TREX-2; it interacts with the NudC chaperone and the dynein/dynactin motor complex and is necessary for mRNA transport [17].

The interaction between TREX-2 and the nuclear pore complex is important for efficient mRNA transport [2,19]. TREX-2 has been shown to interact with TPR (dMegator) and NUP153, which are the components of the nuclear basket in humans [16]. Of these two proteins, TPR is the key structural component of the nuclear basket fibrils, while NUP153 is located at its base [20]. Both components were also found in the nucleoplasm of *Drosophila* cells [21,22]. TPR and NUP153 have been identified as the components responsible for the interaction with TREX-2 in humans [16]. The GANP and ENY2 subunits of the TREX-2 complex are responsible for the interaction with the nuclear pore [16]. It has recently been shown that in humans, TPR, which directly interacts with GANP, plays the main role in TREX-2 binding to the nuclear pore [23]. Suppression of GANP expression leads to relocation of other TREX-2 components from the nuclear pore to the nucleoplasm [16,23].

At the same time, in yeast, the Sus1 subunit of TREX-2 (the homolog of human and *Drosophila* ENY2) together with Sac3 (the homolog of Xmas-2) form the platform for the interaction with Nup1 (the homolog of Nup153) [19,24]. In this way, the existing data show that TREX-2 interaction with the nuclear pore can be organized differently in different organisms.

However, the function of these nucleoporins in the TREX-2-dependent mRNA export in *Drosophila* as well as which TREX-2 subunits interact with the components of the nuclear pore remain unknown. In our work, we addressed these questions using the TREX-2-dependent *hsp70* mRNA export model in *Drosophila*. We have shown that Nup153 and TPR interact with TREX-2 subunits and are indispensable for TREX-2-dependent mRNA export.

## 2. Results

### 2.1. TPR and Nup153 Are Required for TREX-2-Dependent Export of hsp70 mRNA

**Figure 1:**
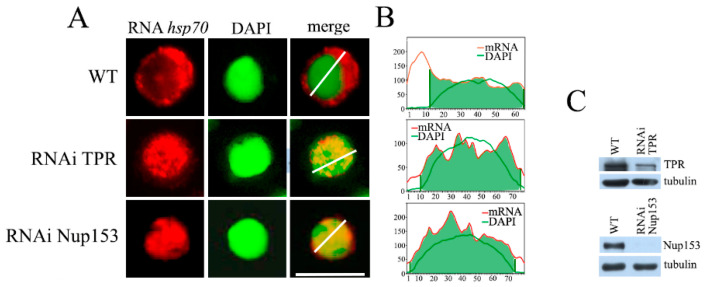
The effect of RNAi-mediated knockdown of TPR and Nup153 on *hsp70* mRNA export. (**A**) The representative examples of the control and TPR and Nup153-depleted cells (at ×1000 magnification). *hsp70* mRNA distribution (red), cell nuclei (green) and their merged images are demonstrated. *hsp70* transcripts were detected by RNA FISH using the (DIG)-labeled strand-specific *hsp70* riboprobes and rhodamine-conjugated anti-DIG antibodies; nuclei were stained with DAPI. Images were re-colored using the Photoshop software v26.9.0 to achieve better visualization. Scale bar, 10 mkm, is shown at the bottom right. (**B**) Quantitative assessment of the fluorescence signals. The signal intensity (*Y*-axis) in each point (*x*-axis) is provided. The distribution of the DAPI signal is shown by the green curve, while the red curve shows the distribution of the *hsp70* mRNA signal. Quantitation was performed using the ImageJ 1.54p software. (**C**) Western blot verification of the specificity of TPR and Nup153 knockdown. The presence of TPR and Nup153 was tested in the control cells treated with the nonspecific dsRNA (GFP) (WT) or with the dsRNA corresponding to TPR and Nup153. Tubulin was used as the loading control.

In the first step, we investigated whether Nup153 and TPR are involved in TREX-2-dependent export in *Drosophila*. We have previously shown that TREX-2 is involved in the export of *hsp70* mRNA and that the knockdown of TREX-2 subunits leads to *hsp70* mRNA being blocked in the nucleus [3]. Therefore, we used RNA FISH to investigate the effects of Nup153 and TPR knockdown on *hsp70* mRNA export. We performed the knockdown of the corresponding nucleoporin in the S2 cells (Figure 1C) and induced the expression of the *hsp70* gene by heat shock.

The obtained results show that the knockdown of both nucleoporins affected the export of *hsp70* mRNA (Figure 1A and Figure S1B). While under normal conditions, mRNA is mostly located in the cytoplasm, and TPR knockdown led to *hsp70* mRNA accumulation in the cell nucleus. Moreover, accumulatedhsp70 mRNA formed foci (Figure 1A). Interestingly, a similar effect of TPR knockdown was observed for the total mRNA in human cells [23]. Nup153 knockdown also led to *hsp70* mRNA redistribution within the cell. Similarly to the case with TPR, we observed mRNA accumulation in the cell nucleus in the form of discrete particles (Figure 1A). The results of the mRNA distribution study are also presented as plots (Figure 1B).

### 2.2. Nup153 Knockdown Alters TPR Localization

**Figure 2:**
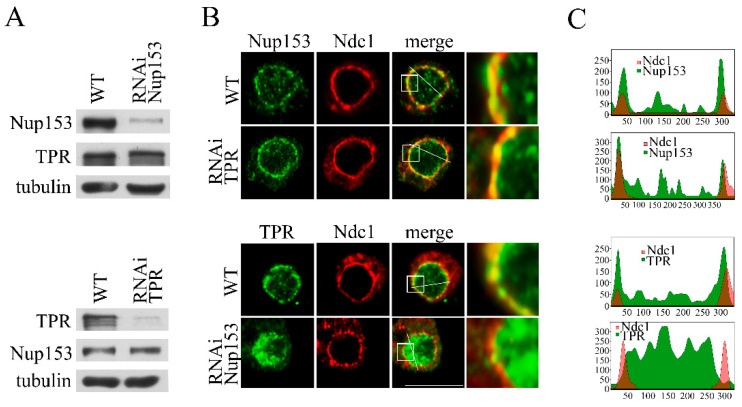
Nup153 affects the distribution of TPR in the cell nucleus. RNAi-mediated knockdown of TPR and Nup153 as well as the control mock RNAi with the GFP region-specific dsRNA were performed in the S2 cells. (**A**) Nup153 and TPR in the control S2 cells (WT) and the S2 cells with TPR or Nup153 RNAi knockdown. Nup153 and TPR were detected in the protein extracts by Western blotting using the corresponding antibodies. Tubulin was used as the loading control. (**B**) Control S2 cells (WT) and the S2 cells with the TPR or Nup153 RNAi knockdown stably expressing dNdc1-mCherry (red) stained with the antibodies against Nup153 or TPR (green), respectively. The white square-framed region of the image was magnified by 5 times and is shown in the panel to the right in each case. Scale bar, 10 mkm, is shown at the bottom right. (**C**) Quantitative assessment of the fluorescence signal. The diagrams show the distribution of the fluorescence signal (red: -mCherry-Ndc1; green: the corresponding antibody; overlapping color - brown) along the white line indicated in the panel B. The x-axis represents the distance in pixels; the y-axis represents the signal intensity in arbitrary units of measurement. Quantitation of fluorescence signal levels was performed using the ImageJ 1.54p software.

In the next step, we checked whether Nup153 knockdown affects the expression levels of TPR and its association with the nuclear pore and, vice versa, whether the knockdown of TPR affects Nup153 since the data obtained with human cells are quite contradictory [16,23,25,26] and this question has never been investigated in *Drosophila*. Our findings demonstrate that TPR knockdown had no effect on Nup153 levels in the cell, with the same observations being made for Nup153 knockdown (Figure 2A).

The knockdown of the TPR nucleoporin did not result in any visible disruption of the Nup153 association with the nuclear pores (Figure 2B,C and Figure S2A). However, the knockdown of Nup153 completely changed the TPR localization. The association of TPR with the nuclear membrane was broken, and the protein’s redistribution to the inner part of the nucleus occurred (Figure 2B and Figure S2B). Thus, the observed effects of Nup153 knockdown on mRNA export may not be direct. The data obtained here point to the important role of TPR in the TREX-2-dependent export of *hsp70* mRNA.

### 2.3. TPR and Nup153 Knockdown Disrupts the Localization of TREX-2 Subunits in the Cell

**Figure 3:**
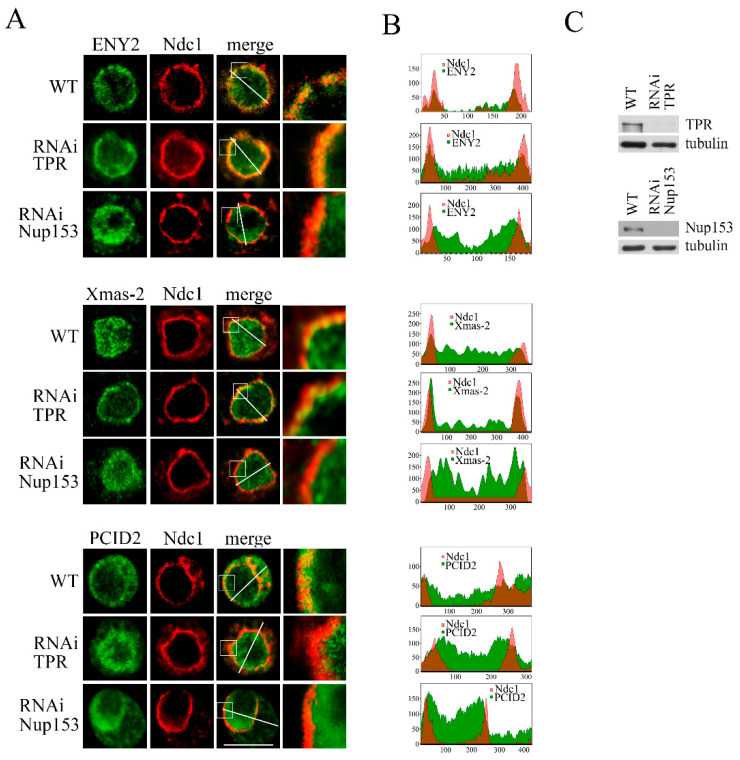
TPR and Nup153 knockdown alters the distribution of the TREX-2 complex subunits in the cell. RNAi-mediated knockdown of TPR, Nup153 or the control mock RNAi with the GFP fragment dsRNA (WT) was performed in the S2 cells. (**A**) Control S2 cells (WT) or S2 cells with TPR or Nup153 RNAi knockdown stably expressing dNdc1-mCherry (red) stained with the corresponding antibodies against ENY2, Xmas-2 or PCID2 (green). The region of the image highlighted with the white square was magnified by 5 times and is shown in the rightmost panel in each case. Scale bar, 10 mkm, is shown at the bottom right. (**B**) Quantitative assessment of the fluorescence signal. The diagrams show the distribution of the fluorescence signal (red: -mCherry-Ndc1; green: the corresponding antibody; overlapping color—brown) measured along the white line indicated in panel A. The x-axis represents the distance in pixels, and the y-axis represents the signal intensity in arbitrary units of measurement. Quantitation of fluorescence signal levels was performed using the ImageJ 1.54p software. (**C**) The specificity of TPR and Nup153 knockdown verified by Western blotting. The presence of TPR and Nup153 was analyzed in the control cells treated with the nonspecific dsRNA (GFP) (WT) or the dsRNA corresponding to TPR and Nup153. Tubulin was used as the loading control.

We further investigated the effects of TPR and Nup153 knockdown on the distribution of TREX-2 subunits within the cell. The knockdown was performed in the S2 cells stably transfected with the nuclear pore channel marker Ndc1 fused to mCherry (Figure 3C).

Under normal conditions, ENY2 was predominantly found in the cell nucleus, being mostly localized in the vicinity of the nuclear pores (Figure 3A and Figure S3).

TPR knockdown did not alter the distribution of ENY2. The knockdown of Nup153 had a pronounced effect, leading to ENY2 accumulation in the nucleoplasm (Figure 3A). Our findings on ENY2 redistribution are also presented as plots shown to the right (Figure 3B).

TPR knockdown affected Xmas-2, leading to the disappearance of the Xmas-2 fraction localized in the nucleus. However, the association of Xmas-2 with the nuclear membrane was retained (Figure 3A,B). On the contrary, Nup153 knockdown led to the disruption of Xmas-2 association with the nuclear membrane and its relocation to the nucleoplasm (Figure 3A,B). Thus, TPR affects Xmas-2 localization in the nucleus, while Nup153 is required for its association with the nuclear pore. It is possible that Xmas-2 relocation to the nucleoplasm reflects the effects of TPR on the Xmas-2 dynamic association with the pore and its shuttling in the nucleoplasm.

The distribution of PCID2 was changed by both TPR and Nup153 knockdown (Figure 3A,B). Normally, PCID2 is found throughout the cell, predominantly in the cytoplasm. The knockdown of both nucleoporins led to its localization in the cell nucleus.

Hence, it can be concluded that both Nup153 and TPR play a role in the TREX-2 interaction with the nuclear pore. Similarly to its effect on TPR, the knockdown of Nup153 leads to the redistribution of all the components of this complex to the nucleoplasm. In contrast, the TPR knockdown only leads to the redistribution of PCID2 to the nucleoplasm.

### 2.4. TREX-2 Has Multiple Contacts with the Nuclear Pore

**Figure 4:**
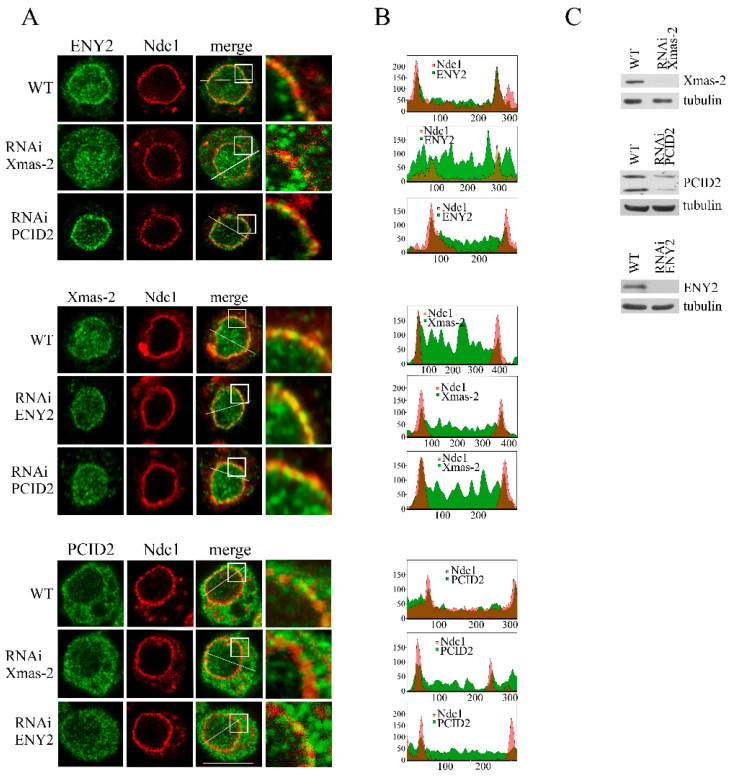
Several TREX-2 subunits are responsible for the complex interaction with the nuclear pore. The RNAi-mediated knockdown of Xmas-2, PCID2 and ENY2 or control mock RNAi with the dsRNA corresponding to a GFP fragment (WT) was performed in the S2 cells. (**A**) S2 cells stably expressing dNdc1-mCherry (red) were stained with the antibodies against ENY2, Xmas-2 or PCID2 (green) in the control (WT) and in the case of Xmas-2, PCID2 or ENY2 RNAi knockdown. The white square-framed region of the image was zoomed in 5 times and is shown in the rightmost panel in each case. Scale bar, 10 mkm, is shown at the bottom right. (**B**) Quantitative assessment of the fluorescence signal. The diagrams show the distribution of fluorescence (red: -mCherry-Ndc1; green: the corresponding antibody; overlapping color - brown) measured along the white line indicated in panel A. The x-axis represents the distance in pixels; the y-axis represents the signal intensity in arbitrary units of measurement. Quantitation of fluorescence signal levels was performed using the ImageJ 1.54p software. (**C**) The specificity of Xmas-2, PCID2 and ENY2 knockdown verified by Western blotting. The presence of the indicated proteins was analyzed in the control cells (WT) treated with the nonspecific dsRNA (GFP) or the dsRNA corresponding to Xmas-2, PCID2 and ENY2. Tubulin was used as the loading control.

Next, we investigated which subunits of the TREX-2 complex are involved in its interaction with NPC. Towards this end, the effects of the knockdown of each TREX-2 subunit on the localization of the other subunits were studied in the S2 cells (Figure 4). The subunits were visualized by immunostaining with the corresponding antibodies.

ENY2 and PCID2 knockdown had no effect on the localization of Xmas-2 (Figure 4A,B and Figure S4). In contrast, the depletion of Xmas-2 had a strong effect on ENY2 localization, with its levels having significantly increased in the nucleoplasm and the cytoplasm of the cells (Figure 4A,B and Figure S5). We observed almost no ENY2 located at the nuclear pores, suggesting that Xmas-2 is required for ENY2 interaction with NPC. The increased ENY2 levels in the cytoplasm could be explained by Xmas-2 knockdown leading to ENY2 being inefficiently imported into the nucleus. It has previously been shown that human ENY2 co-translationally interacts with GANP, the Xmas-2 homolog, and that the two proteins are imported into the nucleus as a complex [27]. Depletion of Xmas-2 or ENY2 did not lead to any change in the localization of PCID2 in the cell, indicating relative autonomy of PCID2 in the TREX-2 complex (Figure 4A,B and Figure S4). The obtained results indicate that TREX-2 has multiple contacts with NPCs which involve its different subunits.

### 2.5. TREX-2 Subunits Interact with TPR и Nup153

**Figure 5:**
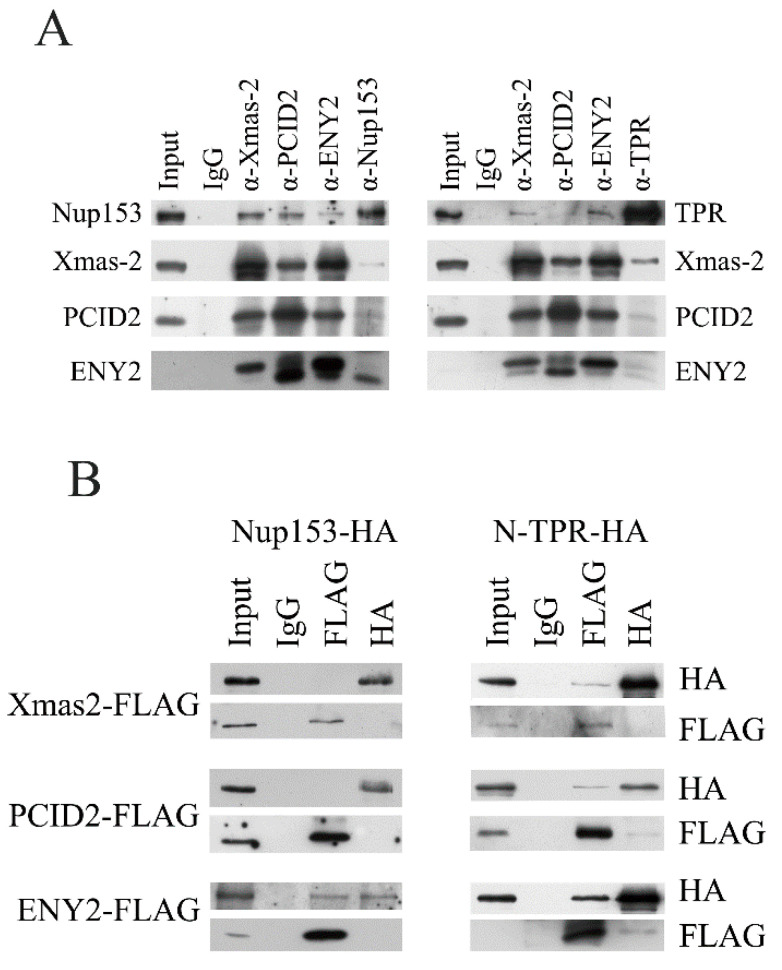
TREX-2 subunits interact with both Nup153 and TPR. (**A**) Immunoprecipitation with the antibodies against Nup153, TPR and TREX-2 subunits. Nuclear extracts obtained from *Drosophila* embryos were treated with DNase and RNase, and immunoprecipitation was performed with the polyclonal antibodies against TREX-2, Nup153, TPR or rabbit immunoglobulin G (IgG), respectively, bound to protein A-Sepharose granules. Equal amounts of the original extract and the proteins bound to the immunosorbent (IP) were separated using SDS-PAGE and analyzed by Western blotting with the above-mentioned antibodies. (**B**) TREX-2 components directly interact with TPR and Nup153. FLAG-tagged Xmas-2, PCID2 and ENY2 were co-expressed in the S2 cells together with HA-tagged Nup153 and TPR. Their interaction was verified in the pull-down assay with the antibodies against FLAG or HA epitopes.

At the next stage, association of TREX-2 with nucleoporins was tested by co-immunoprecipitation from S2 cell extract (Figure 5A).

Nup153 co-precipitated rather efficiently with all TREX-2 subunits, while in the reciprocal experiment, only the ENY2 subunit was co-precipitated with the antibodies against Nup153 at a good level (Figure 5A, left panel). A weak association between TREX-2 and TPR was also observed (Figure 5A, right panel). The Xmas-2 subunit was most efficiently co-precipitated with the antibodies against TPR. Therefore, in co-immunoprecipitation, TREX-2 showed interaction with Nup153 and a weaker interaction with TPR. The strongest association with Nup153 was shown by the ENY2 subunit, and the strongest association with TPR was shown by the Xmas-2 subunit.

Next, we performed pull-down experiments to determine which TREX-2 subunits interact with Nup153 and TPR in the S2 cells (Figure 5B). The subunits of the TREX-2 complex fused with the FLAG epitope were co-expressed in pairs with Nup153 and TPR, which were fused with the HA epitope. To achieve more efficient expression, TPR was split into the N- and C-terminal fragments. The pull-down experiment results demonstrated that the ENY2 subunit interacts with Nup153 (Figure 5B, left panel). The N-terminal fragment of TPR interacted with all three subunits of TREX-2, although only weakly (Figure 5B, right panel). The C-terminal TPR fragment did not interact with TREX-2 subunits (Figure S6). Thus, all subunits of TREX-2 can interact with TPR, while the interaction of TREX-2 with Nup153 occurs via ENY2.

## 3. Discussion

In the present work, we have studied the interaction between TREX-2 and nuclear basket subunits and their role in the TREX-2-dependent mRNA export in *Drosophila*. Our findings demonstrate that both nuclear basket large nucleoporins Nup153 and TPR are required for TREX-2 interaction with the nuclear pore. Their knockdown leads to the impairment of *hsp70* mRNA export and causes *hsp70* mRNA retention in the nucleus, similar to the knockdown of the TREX-2 subunits .

Previously, it has been demonstrated that in human cells, NUP153 is necessary for the interaction between TPR, the main structural component of nuclear basket filaments, and the nuclear pore complex [26]. However, the data obtained by other authors indicated that TPR association with the pore is independent of NUP153 [25]. Our data show that Nup153 is necessary for the TPR association with the nuclear pore complex in *Drosophila* since Nup153 knockdown causes TPR relocation to the nucleoplasm. The relocation of TPR to the nucleoplasm as a result of Nup153 knockdown has previously been demonstrated in human cells [26]. Similarly, redistribution to the nucleus resulting from Nup153 knockdown has also been demonstrated for TREX-2 subunits (Figure 3).

However, this does not mean that TPR is not involved in the interaction with TREX-2, since its knockdown also alters the localization of the TREX-2 subunits: PCID2 remains in the nucleoplasm, while Xmas-2 remains associated with the nuclear pores but its level in the nucleus decreases. The latter indicates that TPR may be involved in the Xmas-2 dynamic interaction with the nuclear pore and that when it is knocked down, the complex cannot relocate to the cell nucleus. The interaction between the TREX-2 subunits and TPR is also confirmed by the results of the pull-down assay. Thus, TPR is involved in a complex system of interactions with TREX-2.

The results of the pull-down assay show that the ENY2 subunit interacts with Nup153. This is in good agreement with the fact that in yeast, Sus1, the ENY2 homolog, and the C-terminal fragment of Sac1 (dXmas-2) form the platform for the interaction with Nup1 (dNup153). In our experiments, the pull-down assay did not reveal any interaction between Xmas-2 and Nup153, which may reflect that dXmas-2 needs ENY2 for interaction. The knockdown of ENY2 in the cells does not lead to the dissociation of Xmas-2 from the nuclear pores since Xmas-2 also interacts with nuclear pores through TPR.

Interestingly, the knockdown of TPR and Nup153 leads to PCID2 accumulation in the cell nucleus. This confirms that PCID2, as it was previously shown, is the shuttle protein which dissociates from the TREX-2 complex at the nuclear pore and enters the cytoplasm together with mRNA molecule [17,18].

Our work shows that in *Drosophila*, both nucleoporins are necessary for TREX-2-dependent export and for the interaction of NPC with TREX-2. It can be suggested that firstly, TREX-2 components interact with the filaments of the nuclear basket, which consist of TPR, and then Xmas-2 together with ENY2 interact with Nup153, which is located at the base of the nuclear basket.

The present work was carried out using the model gene *hsp70*, for which it has been demonstrated that its mRNA export depends on TREX-2 both in *Drosophila* and human [2,3,16]. The *hsp70* mRNA does not undergo splicing. It can be assumed that in the case of intron-containing mRNAs [28], the requirements for certain nucleoporins in the process of mRNA export may slightly differ.

## 4. Materials and Methods

### 4.1. Expression Vectors

The protein-coding sequences of the Xmas-2, PCID2 and ENY2 genes were cloned in frame with the FLAG tag into the pAc5.1/V5-His B vector (Invitrogen, Waltham, MA, USA). The protein-coding sequences of Nup153 and N-TPR (1-1115 aa) were fused to the HA tag and cloned into the pAc5.1/V5-His expression vector (Invitrogen). The protein-coding sequence of Ndc1 was fused to the mCherry tag and was cloned together with the FLAG tag into the pAc5.1/V5-His B expression vector (Invitrogen). The details of the cloning procedures, primers and plasmids used to obtain the constructs are available upon request.

### 4.2. Antibodies

The polyclonal antibodies against Xmas-2, PCID2 and ENY2 obtained earlier in our laboratory were used [3,10,29]. Polyclonal anti-Nup153 antibodies (amino acids 1-350) were raised by immunization of rats with the corresponding His6-tagged protein fragment in our laboratory (Figure S1A). The antibodies against TPR were kindly provided by A. Golovnin. The antibodies against lamin Dmo (obtained by P.A. Fisher) and the antibodies against α-tubulin were obtained from the Developmental Studies Hybridoma Bank established by the NICHD and maintained at the Department of Biological Sciences, University of Iowa. The specific primary antibodies against HA (C29F4) and FLAG M2 (F3165) were purchased from Cell Signaling Technology and Sigma-Aldrich, respectively.

### 4.3. Drosophila Cultured Cell Lines

Drosophila Schneider line 2 (S2) cells were maintained at 25 °C in the Schneider’s Insect Medium (HiMedia, Mumbai, India) containing 10% Fetal Bovine Serum (HyClone, Logan, UT, USA). S2 cells were used to obtain a stable cell line expressing Ndc1-mCherry as the marker of the nuclear pore channel. S2 cells were co-transfected with the desired expression construct and the pCoBlast selection vector (Invitrogen), which bears the gene conferring resistance to blasticidin, at the 20-to-1 molar ratio using the MACSfectin (Miltenyi Biotec, Bergisch Gladbach, Germany) transfection reagent. To select transfectants, S2 cells were grown in the blasticidin-containing S2 medium (25 μg/mL final concentration).

### 4.4. Drosophila Cell Culture Extract

To perform experiments on the FLAG-tagged and HA-tagged protein co-immunoprecipitation from the extract, the respective proteins were co-expressed in the S2 cells for 4 days. For protein extraction, S2 cells were lysed in 10 mM HEPES, pH 7.9, containing 5 mM MgCl2, 0.5% NP-40, 0.45 M NaCl, 1 mM DTT and complete protease inhibitor cocktail (Roche Diagnostics GmbH, Mannheim, Germany). Co-immunoprecipitation was performed as describedin [10] with extract pre-treatment using DNase I (Thermo Scientific, Waltham, MA, USA, 1 U/μL) and RNase (Thermo Scientific, 10 U/μL). Immunoprecipitation was performed using the anti-FLAG^TM^ M2 affinity agarose gel (Sigma, Saint Louis, MO, USA) and the EZviewTM red anti-HA affinity agarose gel (Sigma). The results of immunoprecipitation were revealed by Western blotting with the corresponding antibodies.

### 4.5. Drosophila Embryonic Nuclear Extract

Nuclear extracts from Drosophila embryos were used for immunoprecipitation experiments. Nuclear material was extracted from 0 to 12 h *Drosophila* embryos using 0.42 M ammonium sulfate solution as described previously [29]. For immunoprecipitation experiments, 150 μg of the nuclear extract was diluted with 400 μL of IP buffer (25 mM Tris-HCl, pH 7.9; 10% [vol/vol] glycerol; 0.1% NP-40; 0.5 mM DTT and 5 mM MgCl2) containing 100 mM KCl. Immunoprecipitation was performed as described previously [29] using protein A Sepharose (Sigma) and the corresponding antibodies. The proteins bound to the antibody-protein A Sepharose were washed subsequently with 500 mM KCl and 100 mM KCl-containing IP buffer. The results of immunoprecipitations were revealed by Western blotting with the corresponding antibodies.

### 4.6. RNAi Knockdown in S2 Cells and Transfection Experiments

RNAi procedures were carried out following a previously published protocol [30]. For RNAi, 5–7 μg of dsRNA per 1 × 10^6^ cells was used. dsRNA was synthesized using the Transcript Aid T7 High Yield transcription kit (Thermo Scientific). Expression of the target genes was evaluated by qPCR and Western blot analysis. dsRNA corresponding to a green fluorescent protein (GFP) fragment was used as the negative control. The following pairs of primers were used to amplify dsRNAs:

GFP: 5′-CGACTCACTATAGGGAGACGTAAACGGCCACAAGTTCAGC-3′ and 5′-CGACTCACTATAGGGAGAGATGCCGTTCTTCTGCTTGTCG-3′;

Xmas-2: 5′GAATTAATACGACTCACTATAGGGAGAATGACCTGCACCGTAAG-3′ and 5′-GAATTAATACGACTCACTATAGGGAGACCGGTTGTAGTTCATAG-3′

PCID2: 5′-CGACTCACTATAGGGAGAGTAGGTAGACGGGCTATGTTCG-3′ and 5′-CGACTCACTATAGGGAGACAGTTTGT TGTGAGCATGTGAG-3′;

ENY2: 5′-CGACTCACTATAGGGAGACACTTCCGGCGCAGTTGAT-3′ and 5′-CGACTCACTATAGGGAGAGATTCGTCCTCTGGCTCATCG-3′;

Nup153: 5′-CGACTCACTATAGGGAGAATGGAGGATGCACAGGAGCA-3′ and 5′-CGACTCACTATAGGGAGACAGCAGGCGTGGATGACAAA-3′;

TPR: 5′-CGACTCACTATAGGGAGACCGCTTGGAGTCCGCTGAAG-3′ and 5′-CGACTCACTATAGGGAGACTAGGGGAATCGTCCTCCGTTG-3′.

Transient transfection of S2 cells was performed using the MACSfectin (Miltenyi Biotec) transfection reagent according to the manufacturer’s instructions.

### 4.7. RNA FISH

To perform RNA FISH of individual genes, S2 cells were fixed with 3.7% PFA on glass slides for 20 min. After fixing and washing, S2 cells were subjected to a series of gradual dehydration and rehydration using EtOH, followed by Proteinase K (50 μg/mL) treatment in 1× PBS at 37 °C for 5 min. The reaction was stopped by adding Glycine (2 mg/mL) in 1× PBS. Then, S2 cells were fixed again for 20 min, washed with PBST (1× PBS + 0.1% Tween20) and incubated with the denatured (80 °C, 3 min) digoxigenin (DIG)-labeled strand-specific *hsp70* riboprobes (2 ng/μL) in the buffer containing 50% formamide, 5× SSC, 0.1% Tween20, 100 μg/mL salmon sperm DNA and 50 μg/mL heparin at 42 °C for 16h. The hsp70 probe corresponded to a region within the ORF and was obtained using a pair of primers (5′-CTACTCCTGCGTGGGTGTCTAC-3′ and 5′-TGTCGTTCTTGATCGTGATGTTC-3′) and the DIG RNA labeling kit (SP6/T7) (Roche Diagnostics GmbH, Mannheim, Germany). After hybridization, S2 cells were washed with 2× SSC and treated with RNase A (20 μg/mL) in the buffer containing 500 mM NaCl, 10 mM Tris (pH 8.0) and 1 mM EDTA at 37 °C for 30 min. S2 cells were then washed with SSC according to the following protocol: wash 2 times for 15 min—2× SSC at 37 °C; wash 2 times for 15 min—1× SSC at 37 °C; and wash 2 times for 30 min—0.1× SSC at room temperature. S2 cells were incubated in the blocking solution (2× BR + 2× SSC) in a humid chamber for 2h and then stained for 1h with rhodamine-conjugated anti-DIG antibodies (Roche Diagnostics GmbH, Mannheim, Germany) diluted at 1:50. After hybridization, S2 cells were washed according to the following protocol: 3 times for 15 min—1× PBS + 0.1% Tween20 + 0.3% Triton X-100 and 2 times for 15 min—1× PBS + 0.5% Tween20. To visualize DNA, S2 cells were incubated in PBS containing 0.2 μg/mL DAPI. Finally, S2 cells were dehydrated in EtOH (70–90–100%, 5 min), dried and mounted with Vectashield (Vector laboratories, Newark, CA, USA). The results were checked in the Leica DMi8 inverted fluorescence microscope equipped with the HC PL Fluotar 63×/1.30 oil immersion objective lens. The resulting images were analyzed using the ImageJ 1.54p software [31].

### 4.8. Immunostaining Experiments and Microscopy

In the immunostaining experiments, the *Drosophila* S2 cell line with the stable expression of Ndc1-mCherry was used for nuclear pore complex visualization. For these experiments, the S2 cells were grown on coverslips. Cells were washed twice with 1× PBS, fixed in 3.7% PFA for 10 min, permeabilized in 0.2% Triton X-100 for 5 min and blocked with 3% milk/1× PBS for 10 min. Primary antibodies were diluted to working concentrations with 3%milk/1× PBS, and the slides were incubated with them for 30 min at room temperature in a humid chamber. Rabbit antibodies against Xmas-2, PCID2 and ENY2 and rat antibodies against Nup153 and TPR were used as the primary antibodies. Cy3-conjugated goat anti-rabbit IgG antibodies (AS007) from ABclonal (Woburn, MA, USA) and Alexa Fluor 488-conjugated goat anti-rat IgG antibodies (A48262) from Invitrogen were used as the secondary antibodies. After washing, cells were mounted with the Mowiol mounting media (Sigma). The slides were studied using the Nikon AX (Tokyo, Japan) confocal microscope (Plan Apo 100×/1.45 oil immersion objective) or the Leica STELLARIS (Wetzlar, Germany) 5 confocal microscope (objective: HC PL APO CS2 63×/1.40 oil) or the Zeiss LSM 780 confocal laser scanner microscope (Plan-Apochromat 63×/1.40 oil immersion objective). The resulting images were analyzed using the ImageJ 1.54p software [31].

## Data Availability

All data are included in the article.

## Supplementary Materials

The following supporting information can be downloaded at https://www.mdpi.com/article/10.3390/ijms26178595/s1.
