# Supplementary material for: Nup153 and TPR/Megator Interact with TREX-2 Subunits and Are Essential for TREX-2-Dependent Nuclear Export of hsp70 mRNA in Drosophila
Source: Int J Mol Sci. 2025 Sep 4;26(17):8595. doi: 10.3390/ijms26178595 (PMC12429265; doi:10.3390/ijms26178595)
Supplement: Supplementary file 1 [file ijms-26-08595-s001.zip › Supplementary/Supplementary_legends.pdf]

Figure S1. The effect of RNAi-mediated knockdown of TPR and Nup153 on *hsp70* mRNA export. **(a)** Affinity-purified polyclonal antibodies raised in rats specifically recognize Nup153 in Western blot analysis. The *Drosophila* S2 cells extract was fractionated by SDS PAGE and the membrane was cut into separate strips which were incubated with preimmune serum (PI), anti-Nup153 serum (Im), and anti-Nup153 affinity-purified polyclonal antibodies (Ab). Antiserum was raised against 1–350 amino acids of Nup153. **(b)** The representative examples of the control and TPR and Nup153-depleted cells. *hsp70* mRNA distribution (red), cell nuclei (green), and their merged images are demonstrated. *hsp70* transcripts were detected by RNA FISH using the (DIG)-labeled strand-specific *hsp70* riboprobes and rhodamine-conjugated anti-DIG antibodies; nuclei were stained with DAPI. Images were re-colored using the Photoshop software to achieve better visualization. The large fields are shown.

Figure S2. Nup153 affects the distribution of TPR in the cell nucleus. RNAi-mediated knockdown of TPR and Nup153 as well as the control mock RNAi with the GFP region-specific dsRNA were performed in the S2 cells. **(a, b)** Control S2 cells (WT) and the S2 cells with the TPR **(a)** or Nup153 **(b)** RNAi knockdown stably expressing dNdc1-mCherry (red) stained with the antibodies against Nup153 or TPR (green), respectively. Nuclei were stained with DAPI (cyan). The large fields are shown.

Figure S3. TPR and Nup153 knockdown alters the distribution of the TREX-2 complex subunits in the cell. RNAi-mediated knockdown of TPR, Nup153 or the control mock RNAi with the GFP fragment dsRNA (WT) were performed in the S2 cells. Control S2 cells or S2 cells with TPR or Nup153 RNAi knockdown stably expressing dNdc1-mCherry (red) stained with the corresponding antibodies against ENY2, Xmas-2 or PCID2 (green). Nuclei were stained with DAPI (cyan). The large fields are shown.

Figure S4. The localization of Xmas-2 and PCID2 within the cell is unaffected by the knockdown of other components of the TREX-2 complex. RNAi-mediated knockdown of PCID2, Xmas-2, ENY2 or the control mock RNAi with the GFP fragment dsRNA (WT) were performed in the S2 cells. S2 cells stably expressing dNdc1-mCherry (red) were stained with the antibodies (green) against Xmas-2 or PCID2 in the control (WT) and in the case of ENY2, PCID2 or Xmas-2 RNAi knockdown. Nuclei were stained with DAPI (cyan). The large fields are shown.

Figure S5. Xmas-2 knockdown affects the localization of ENY2 in the cell. The RNAi-mediated knockdown of Xmas-2 or control mock RNAi with the dsRNA corresponding to a GFP fragment (control) were performed in the S2 cells. S2 cells stably expressing dNdc1-mCherry (red) were stained with the antibodies against ENY2 (green) in the control and in the case of Xmas-2 RNAi knockdown. The large fields are shown. Quantitative assessment of the fluorescence signal for five cells in each of the two fields is presented in the right panel. The diagrams show the distribution of fluorescence (red: -mCherry-Ndc1; green: the anti-ENY2 antibody) measured along the white line indicated in the left panel. The x-axis represents the distance in

pixels; the y-axis represents the signal intensity in arbitrary units of measurement. Quantitation of fluorescence signal levels was performed using the ImageJ software.

Figure S6. TREX-2 subunits do not interact with the C-terminal region of TPR. FLAG-tagged Xmas-2, PCID2 and ENY2 were coexpressed in the S2 cells together with the HA-tagged C-terminal fragment of TPR. Their interaction was verified in the pull-down assay with the antibodies against FLAG or HA epitopes.
