# Supplementary figures and images for: Nup153 and TPR/Megator Interact with TREX-2 Subunits and Are Essential for TREX-2-Dependent Nuclear Export of hsp70 mRNA in Drosophila
Source: Int J Mol Sci. 2025 Sep 4;26(17):8595. doi: 10.3390/ijms26178595 (PMC12429265; doi:10.3390/ijms26178595)

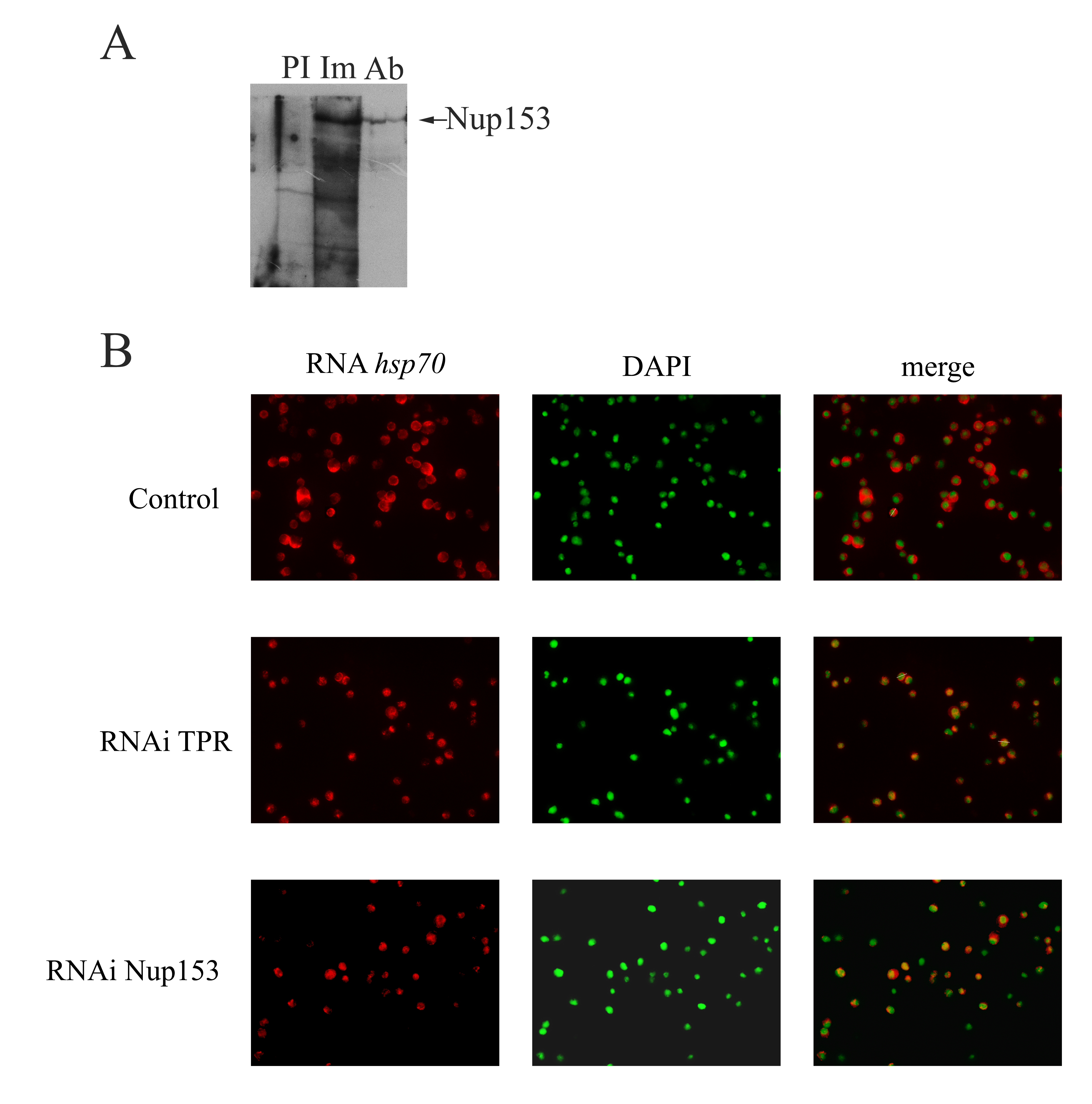

Supplement: Supplementary file 1 [file ijms-26-08595-s001.zip › Supplementary/Figure S1.tif]

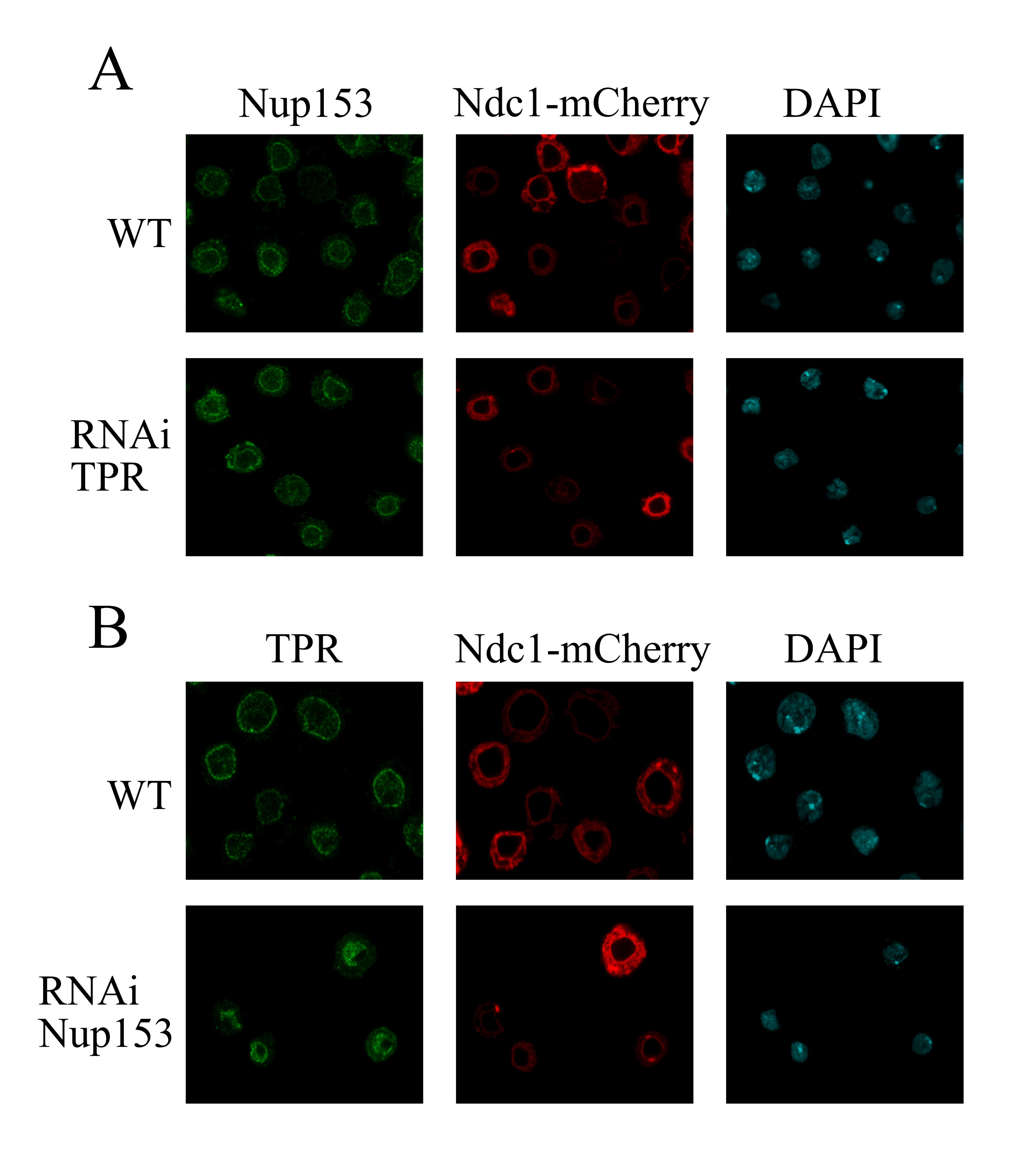

Supplement: Supplementary file 1 [file ijms-26-08595-s001.zip › Supplementary/Figure S2.tif]

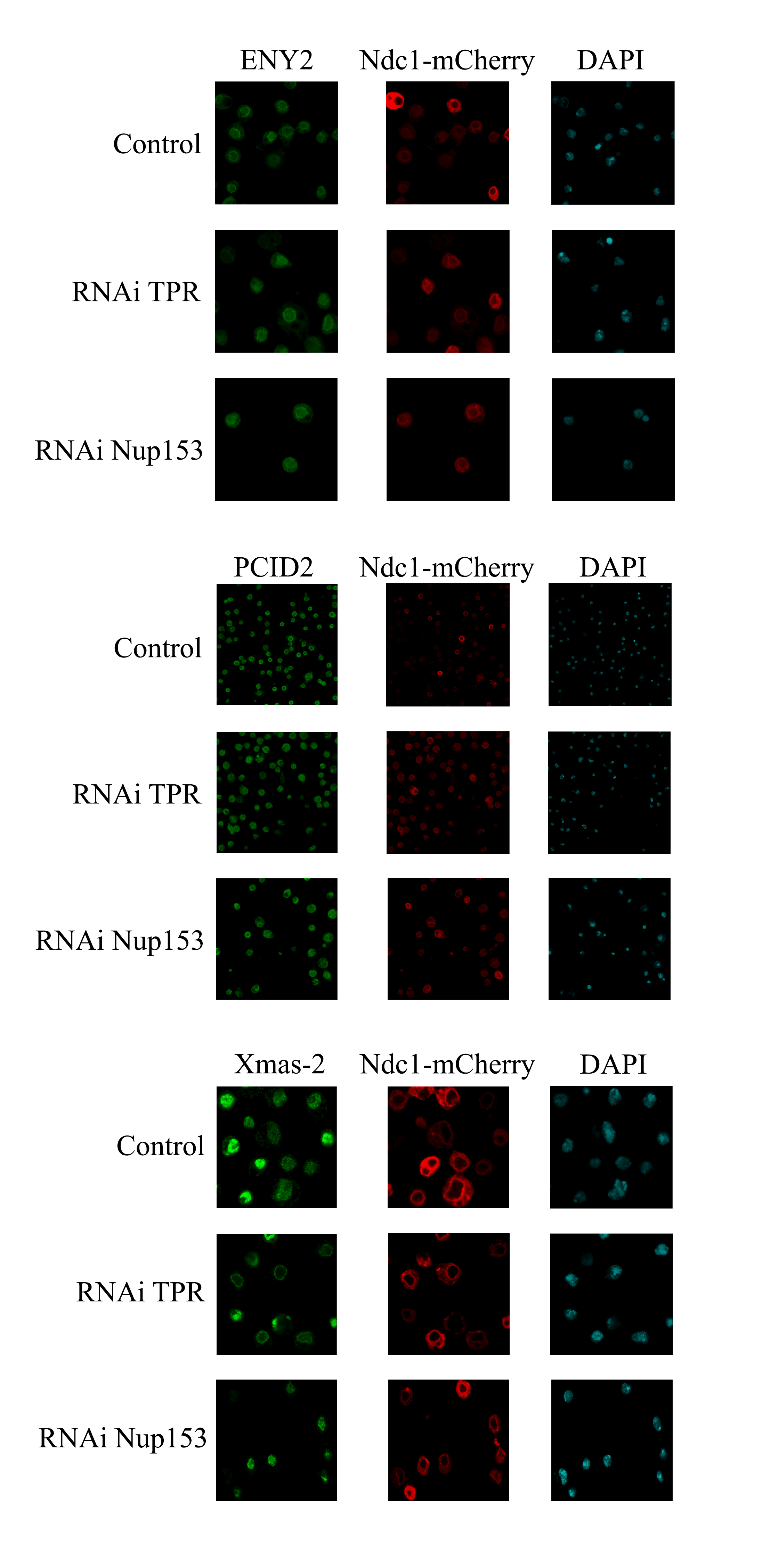

Supplement: Supplementary file 1 [file ijms-26-08595-s001.zip › Supplementary/Figure S3.tif]

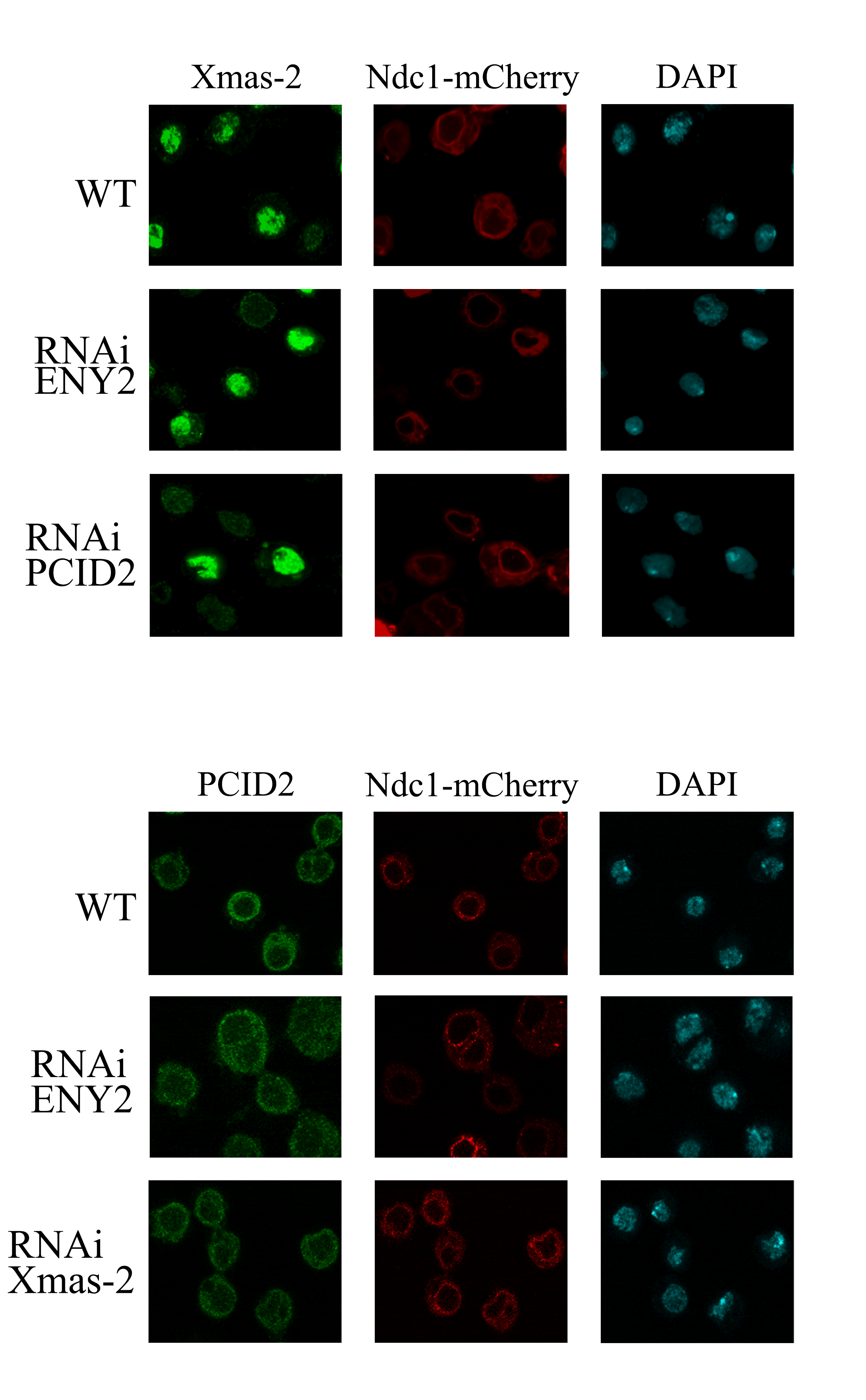

Supplement: Supplementary file 1 [file ijms-26-08595-s001.zip › Supplementary/Figure S4.tif]

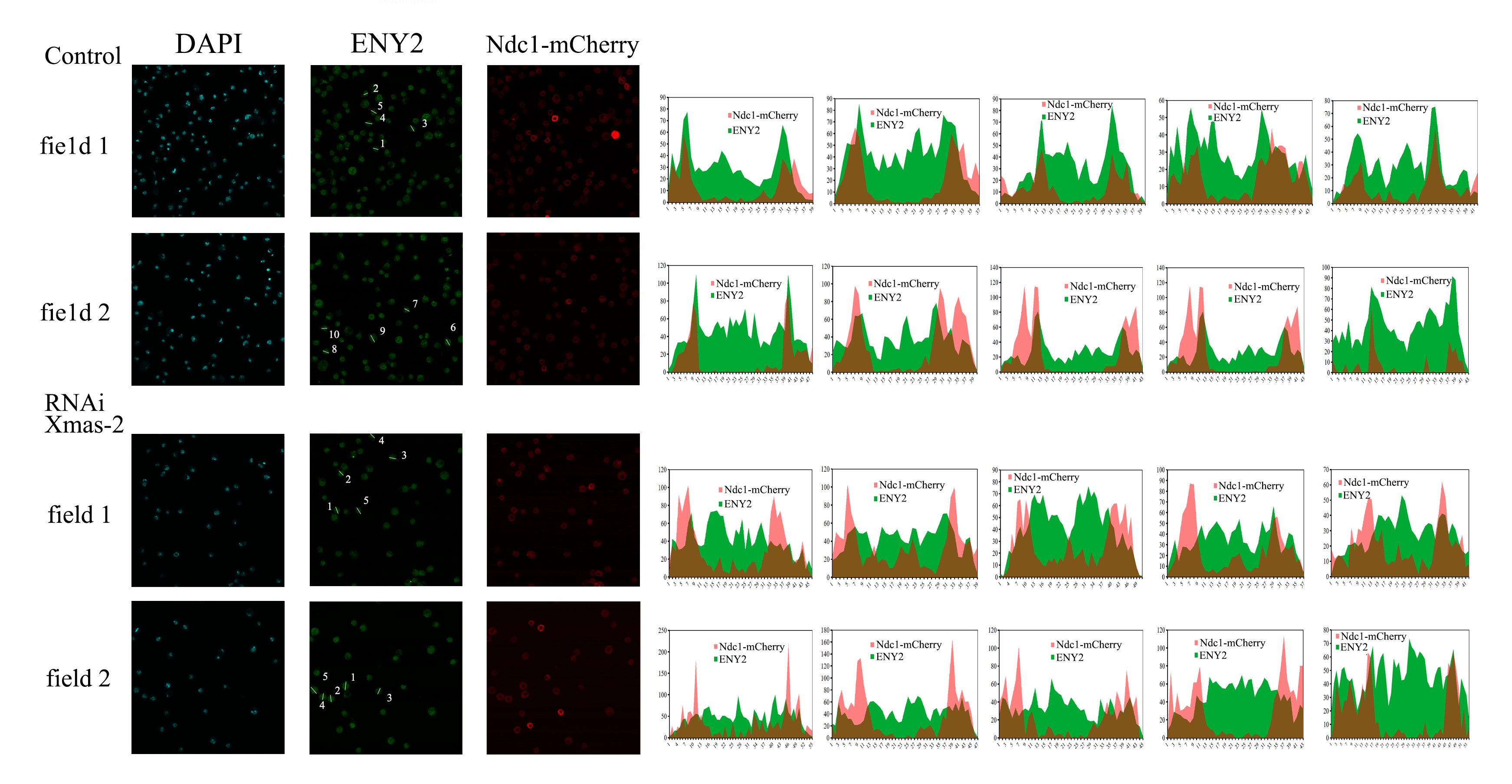

Supplement: Supplementary file 1 [file ijms-26-08595-s001.zip › Supplementary/Figure S5.tif]

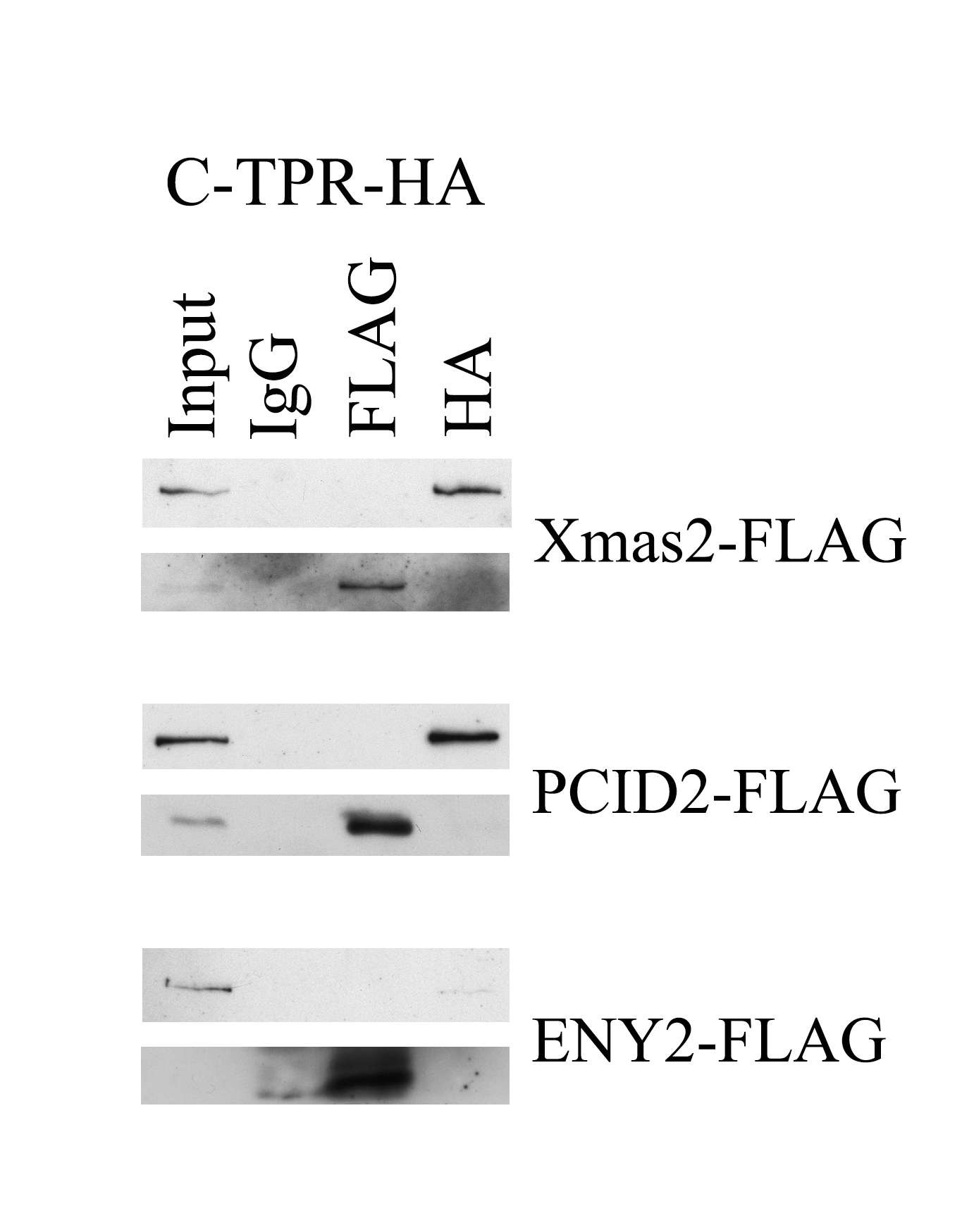

Supplement: Supplementary file 1 [file ijms-26-08595-s001.zip › Supplementary/Figure S6.tif]
